# COVID19: an announced pandemic

**DOI:** 10.1038/s41419-020-02995-9

**Published:** 2020-09-24

**Authors:** Sara Platto, Tongtong Xue, Ernesto Carafoli

**Affiliations:** 1 China Biodiversity Conservation and Green Development Foundation, Beijing, China; 2 Department of Biotechnology, College of Life Sciences, Jianghan University, Wuhan, China; 3 Venetian Institute of molecular Medicine, University of Padova, Padova, Italy

**Keywords:** Ecology, Viral infection

## Abstract

A severe upper respiratory tract syndrome caused by the new coronavirus has now spread to the entire world as a highly contagious pandemic. The large scale explosion of the disease is conventionally traced back to January of this year in the Chinese province of Hubei, the wet markets of the principal city of Wuhan being assumed to have been the specific causative locus of the sudden explosion of the infection. A number of findings that are now coming to light show that this interpretation of the origin and history of the pandemic is overly simplified. A number of variants of the coronavirus would in principle have had the ability to initiate the pandemic well before January of this year. However, even if the COVID-19 had become, so to say, ready, conditions in the local environment would have had to prevail to induce the loss of the biodiversity’s “*dilution effect”* that kept the virus under control, favoring its spillover from its bat reservoir to the human target. In the absence of these appropriate conditions only abortive attempts to initiate the pandemic could possibly occur: a number of them did indeed occur in China, and probably elsewhere as well. These conditions were unfortunately present at the wet marked in Wuhan at the end of last year.

## Facts

• The present COVID-19 pandemic has now spread to more than 200 countries.

• The COVID-19 infection was present in China, and even in Europe, at the end of 2019, i.e., well before its explosion in Wuhan in January of this year.

• At the beginning, it had remained confined in the form of small outbursts, that failed to spread in a significant way.

• The explosion in Wuhan at the end of January 2020 might have been caused by the combination of unfavorable events, chiefly the “*amplification effect*” caused by the presence of the virus reservoir, and viable animals’ hosts at the wet-market.

• Once the COVID-19 infection spread to Europe and the Americas it acquired an enormously increase transmissibility.

## Open questions

• Why did previous similar coronavirus infections, e.g., SARS-CoV, fail to develop to pandemic dimensions, whereas that of COVID-19 was able to do it?

• Is there an obligatory intermediate host in the transmission of COVID-19 from bat to humans?

• Is there a decisive factor that must be satisfied by coronaviruses to successfully attack human cells?

• Why did the COVID-19 infection became so more transmissible from human-to-human once it reached Europe and the Americas?

## Preamble

Globalization and ecological disruption appear to be associated with newly emerging infectious diseases, and with reemerging infections previously thought to be under control, leading to what has been defined as *a new epidemiological transition*^1–3^. Anthropogenic changes of forest habitats can reduce biodiversity and bring people into closer contact with wildlife, increasing the risk of zoonotic disease transmission^4,5^. The biological and ecological system changes can be plausibly considered among the drivers that allow pathogens to successfully cross-over from animal to human, precipitating the emergence of new diseases^5–7^.

Biodiversity has been linked to reduced pathogen transmission through the so called *dilution effect*, which occurs when in a niche there is a variety of host species that negatively affect pathogen persistence by acting as “*buffering species*”^8,9^. Biodiversity loss may increase zoonotic and anthropogenic pathogen exchange by forcing species into atypical ecological interaction that facilitate transmission^5^. This latter condition could be easily encountered in the wet-markets across Asia.

Wet-markets are places where food products and live animals are sold. In general, they are located in the cities, close to residential areas, thus allowing frequent contacts between humans and live food animals^10^. In Southern China, there is the habit of consuming as food or Traditional Chinese medicine products a wide range of exotic animals such as civet cats (*Paguma larvata*), raccoon dogs (*Neyctereutes procyonoides*), and different species of bats^10^. Chinese New Year is the highest season when a lot of these animals are sold, and it coincides with the winter period characterized by the high incidence of respiratory tract infectious diseases^10^.

In the wet-markets, live animals of different species are crowded in small spaces with poor or nonexistent hygienic conditions, and with a huge dispersal of excrement probably full of potential hazards for human health^10^. This environment could be considered an altered ecosystem, or better, an *amplification effect* zone, where an high density of pathogens’ reservoirs and sensible hosts are forced into atypical interactions, and where the *dilution effect* found in nature is abolished, with higher possibility of the development of outbreaks. In this *altered ecosystem*, humans play the part of susceptible hosts who can be infected either indirectly through an intermediate host, or directly from the reservoir. In the latter option, humans circulating in the wet-markets could become intermediate hosts of their own population. This point will be discussed in more detail later on.

Once arrived in the new environment (human or intermediate host), pathogens such as viruses may acquire new genes or modify existing genes e.g., by genetic recombination: in coronaviruses the rate is of the order of 1 per 10,000 nucleotides, which makes these RNA viruses especially conducive to host switching. In this way, virus variants with higher transmissibility or pathogenicity will emerge^10^. Initially, there could be repeated transmissions of the virus from animals to humans, without a human-to-human transmission, that are defined as *viral chatters*^11^. The *amplification effect* of the wet-markets could increase the rate of the *viral chatters* boosting the diversity of the virus variants, leading the pathogens to adapt to humans, and favoring their spread in the population. If the *amplification effect* is absent, only limited outbreaks are likely to occur, which will remain undetected or confined to remote locations from which the large scale spreading of the infection will not be possible. This is would appear to have repeatedly happened to the COVID-19 prior to the Wuhan explosion.

## COVID-19

**Fig. 1:**
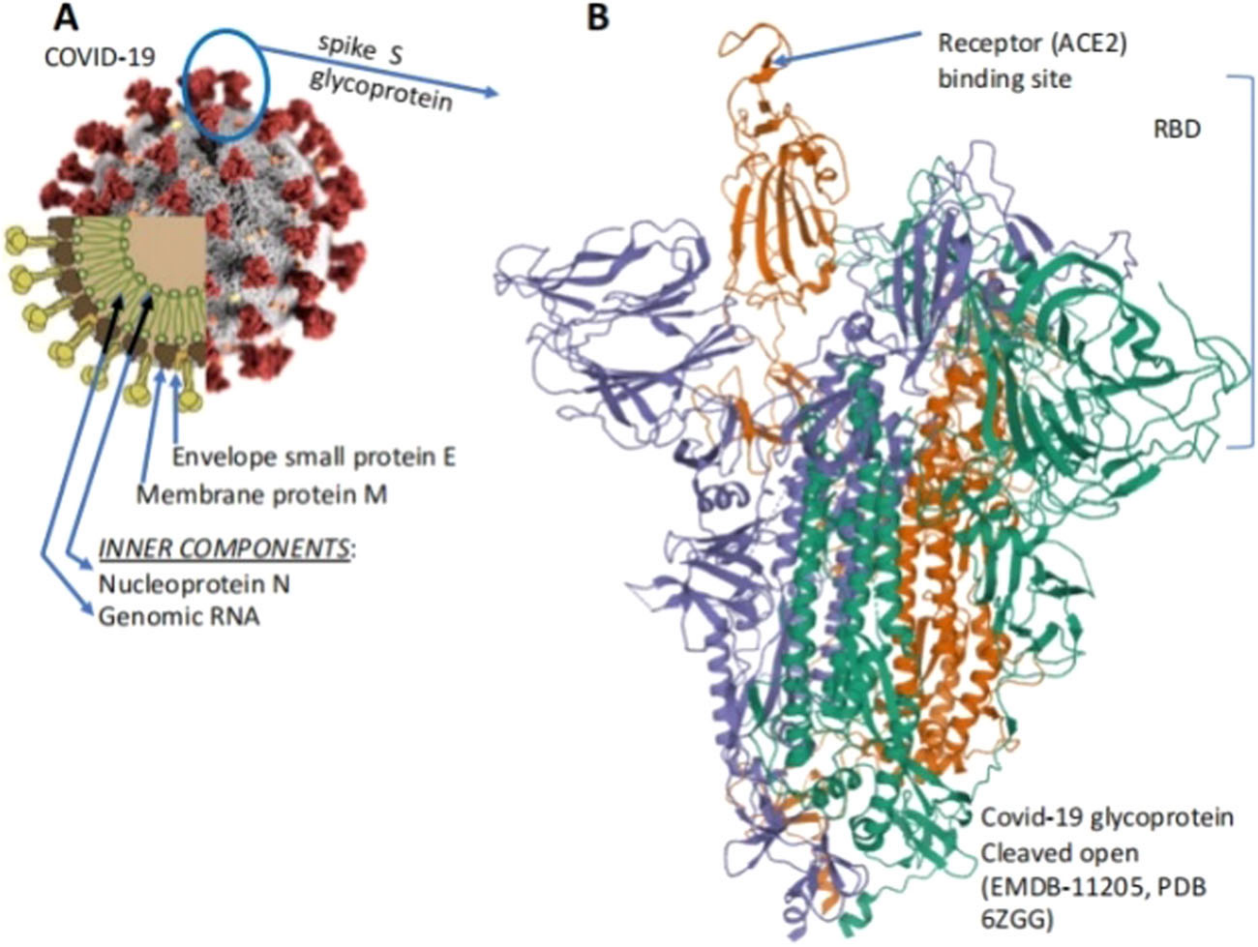
The coronavirus with the Spike trimeric glycoprotein projections from the surface. **a** The schematic portion of the Figure (lower left) shows the structural E and M structural proteins of the membrane, the protruding trimeric structural protein, the structural protein N that binds the RN, and the RNA genome. **b** the 3-D structure of the S-protein with the receptor binding domain (RBD) with the domain that binds the ACE 2 receptor.

**Fig. 2:**
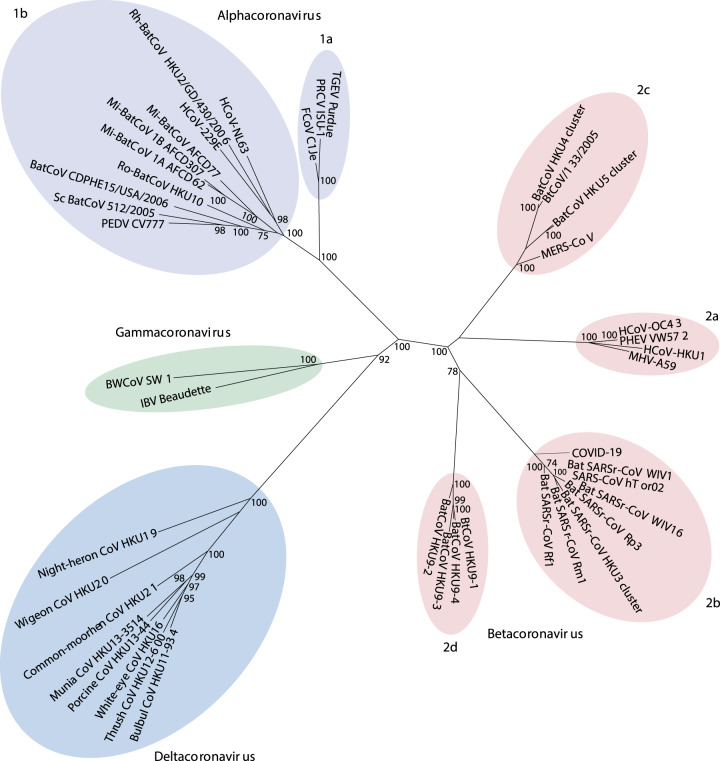
Philogenetic relationships in the Coronaviridae subfamily from the Coronavirinae family. The viruses in the subfamily group into four genera: Alphacoronavirus, Betacoronavirus, Gammacoronavirus, and Deltacoronavirus, based on their phylogenic relationshios and genomic structures; the alphacoronaviruses and betacoronaviruses infect only mammals and usually cause respiratory syndromes in humans, the gammacoronaviruses infect avian species and the deltacoronaviruses infect both birds and mammals. The highly infective viruses SARS-CoV and MERS-CoV are betacoronaviruses, the COVID-19 virus is not listed among the betacoronaviruses because it was not yet known and classified in early 2019. Other details are found in Cui et al. 2019 (Figure modified from Cui et al.)^31^.

COVID-19 (Fig. 1) is a member of the Coronavidae (CoV) subfamily of the Coronavirinae family, which belongs to the order Nidovirales. The subfamily comprises about 40 varieties of single stranded RNA-viruses residing in bats and wild birds, which can evolve to infect humans and non human mammals and birds (Fig. 2). Because of their ability to recombine, mutate, and infect multiple species and cell types, coronaviruses keep emerging and evolving, causing human and veterinary outbreaks^12–14^. The seven common types of human coronaviruses are the following:

229 E (alpha coronavirus)

HKU1 (beta coronavirus)

MERS CoV (beta coronavirus)

NL63 (alpha coronavirus)

OC43 (beta coronavirus

SARS CoV (beta coronavirus)

COVID19 (SARS CoV-2) (beta coronavirus)

**Fig. 3:**
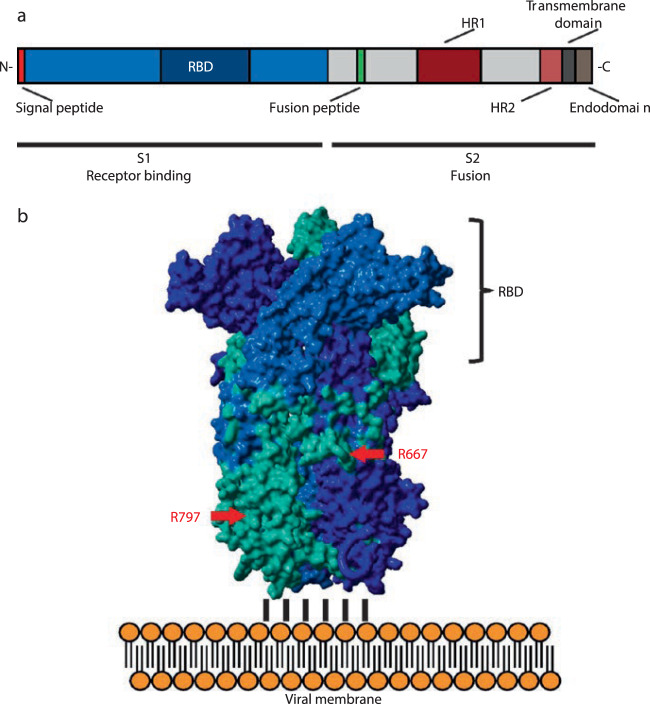
A representation of the structure and domain organization of the spike glycoprotein of the coronavirus, which is formed by the two subunits S1 and S2. At the N-terminus of subunit S1 a signal peptide permits the insertion of the nascent protein into the secretory pathway of the host cell. The S1 subunit contains the receptor binding domain (RBD) that attaches the S protein to the ACE2 receptor of the host cell. The S2 subunit contains the membrane fusion complex (fusion peptide, heptad repeats HR 1 and HR2), anchors the S2 subunits to the viral membrane with its transmembrane domain, and interacts with the viral ribonucleoprotein complex through its endodomain. (modified from Hoffmann et al.)^17^.

The COVID-19 (Fig. 1a) contains four structural proteins that are encoded by open reading frames (ORF) at the 3′end of the RNA genome, and 16 accessory proteins (nsp 1 to nsp 16), which are encoded by other ORFs at its 5′end. The structural proteins E and M form the envelope of the virus, the N protein nucleocapsid binds the viral RNA, and the S glycoprotein interacts with the receptor of the target cells favoring the virus penetration into them. During the SARS-CoV outbreak of 2002–2003, this receptor was identified as the angiotensin-converting enzyme 2^15^ (Fig. 1b). The nascent S protein has two subunits, S1 and S2^16^. The S protein is modified in the endoplasmic reticulum (ER) of the host cell with N-linked glycans that would protect it against neutralizing antibodies^16–18^. After passing the ER quality control, the S protein is transported to the surface of the virus and is incorporated into the viral membrane where it is cleaved (primed) into two segments: (1) the N-terminal S1 segment contains a signal peptide and the receptor binding domain (RBD) that interact with the host cell receptor (Fig. 3)^18^; (2) the S2 segment, which anchors the S protein to the viral membrane, and contains the fusion peptide which mediates the fusion of the viral membrane with the plasma membrane of the target cell. A loop subdomain (RBM) in the RBD contacts directly the receptor^19^.

**Fig. 4:**
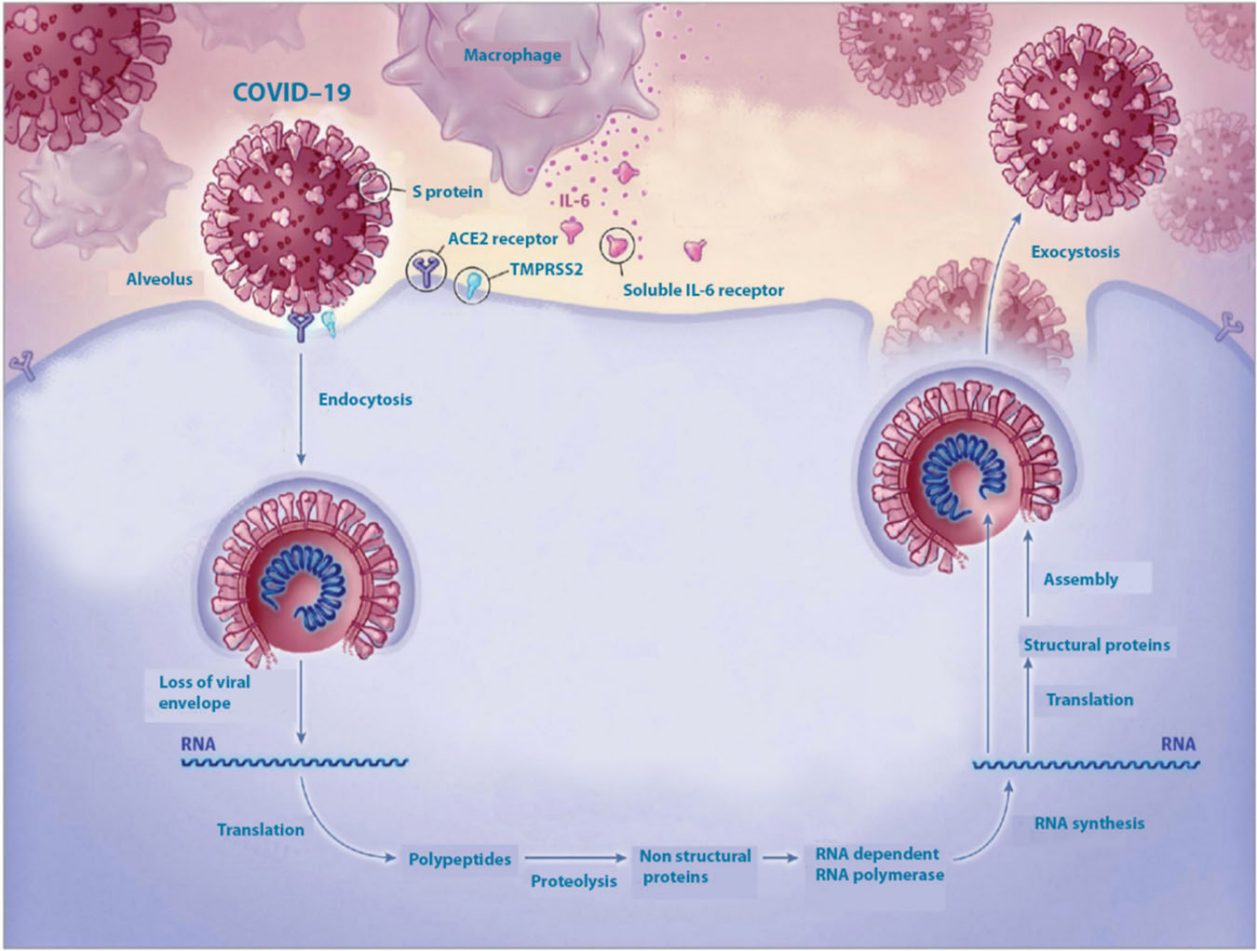
Invasion of the target cell (a pulmonary alveolar cell) by the virus. Liberation of the viral RNA genomei, its translational activity that produces a number of proteins including interleukin 6, and replicates itself. Other details in the text. (modified from the Web).

The cleavage of the S protein is performed by proteases of the host cell, both endosomal, cathepsins B and L, and the transmembrane serine protease TMPRSS2, which appears to predominate^20^. Another protease, the furin is also active in the cleavage of the S-protein, possibly preparing it for the cleavage by TMPRSS2^20^. The two segments of the cleaved S protein remain bound non-covalently, and the binding of the RBD of S1 to the receptor weakens the non-covalent interactions between the two segments. This step exposes the fusion peptide within the S2 segment, promoting its insertion into the cell membrane and the eventual internalization of the virus. The virus can also become internalized by endocytosis, i.e., the membrane of the target cell folds around it to form an endosome. The endosome eventually fuses with the lysosomes, where the hydrolases uncoat the virion liberating its RNA genome in the cytoplasma (Fig. 4). The RNA genome is translated in polyproteins, among which proteases cleave them into non structural proteins (nsp 1–16), of which the nsp 12 is the RNA-dependent RNA polymerase that replicates the RNA genome and catalyzes the synthesis of the viral structural proteins.

Coronavirus genomes (26–32 kilobases) are the largest known RNA genomes, their expansion being due to the acquisition of specific enzyme functions that counter the otherwise very high error frequency of the RNA polymerase (see Box 1). The nsp12 is the central enzyme of the replicase gene in all RNA viruses. It contains the canonical RNA- dependent RNA polymerase motifs in its C-terminal portion, and catalyzes the synthesis of the viral RNA. Its function requires accessory proteins (nsp 7 and nsp 8). The replicase complex of the coronaviruses has special properties such as the strong influence of the accessory proteins (nsp) on its behavior, the high RNA recombination frequency, and the complexity of the subgenomic mRNA synthesis^21^. The 3D structure of the RNA polymerase has now been solved in two variants: one in a complex with the nsp7, and nsp8 proteins; and one in a complex with a nsp 8 monomer^22^. The 3D structure of the polymerase has also been solved in a complex with one of its inhibitors, remdesivir: a phosphoramidate adenosine analog that is processed within the cell to a nucleoside triphosphate derivative that can become integrated into the viral RNA^23^. Once reconstituted, the virion eventually leaves the host cell by exocytosis to attack neighboring targets, i.e., macrophages and T-lymphocytes. One of the structural proteins of the virion, nucleocapsid protein N, catalyzes the synthesis of Interleukin 6 (IL-6) by binding to NF-kB on the promoter of the IL-6 gene. IL-6 is the principal actor in the cytokine storm triggered by the coronavirus^24^.

Box 1 The RNA synthesis and processing function—the *replicase gene*—occupy about two thirds of the genome. Its tracslation products are two large polyproteins, which are cleaved to a number of non structural proteins (nsp 1 to 16). The cleavage is performed by two cysteine proteases, a chymotrypsin-like protease (3CLpro), and a papain-like protease (PLpro), which is the target of interesting inhibitors. In addition to the RNA dependent RNA polymerase (nsp 12) the replication and transcription process (the replicase gene) also contains the helicase (nsp 13) and a number of other nsps that act on the mRNA. Together with other nsps, all these proteins constitute the “*nps interactome*”.

## The puzzle of the direct spillover

The hypothesis of a direct spillover of coronaviruses from an animal reservoir to humans had been already proposed after the SARS-CoV epidemic^25–27^. Since 2005, novel coronaviruses, termed SARS-like-CoV, had been detected in horseshoe bats of the genus *Rhinolophus* from a wide geographic range in China that included the provinces of Guangxi, Hubei, Shangong, Guizhou, Shaanxi, and Yunnan, and Hong Kong^25,28–30^ (see Box 2). At variance with SARS-CoV, all SARS-like-CoV encountered initially were unable to use ACE2 receptors as entry mechanisms in the cells. Therefore, these types of CoVs were not considered able to cause a direct infection in humans, unless they acquired new genes via recombination in other host populations in order to develop new tissue tropism or virulence^30^.

In 2013, diverse SARS-like-CoVs were discovered in a single colony of *Rhinolophus sinicus* in Yunnan Province (China). The colony contained CoVs with all genetic diversity encountered in other locations in China^25^, and the viral strains presented all genetic elements needed to form SARS-CoV^31^. During the same study, a new isolate of SARS-like-CoV, termed W1V1, was also identified with 95% nucleotide sequence identity, to that of human SARS-CoV, i.e., higher than in other SARS-like-CoVs, and able to use ACE2 receptors of different origin as cell entry path^25^. The new isolate was indeed able to replicate efficiently in ACE2-expressing cells, and to grow in human alveolar basal epithelial cells. Its close genetic relation to human SARS-CoV was confirmed by the neutralization effect of convalescent SARS patients sera on WIV1^25^. The high genetic diversity of SARS-like-CoVs within this bat colony was mirrored by high phenotypic diversity in the differential use of ACE2 by different strains. The identification of WIV1-CoV, and its ability to use ACE2 orthologs posed a warning for possible reemergence of a CoV outbreak^25^. On the basis of this premise, Menachery et al.^26^ used circulating bat coronaviruses to construct chimeric viruses that used spike W1V1. They found that viruses using the WIV1-CoV spike protein replicated in primary human airway epithelial cell cultures without further spike adaptation^26^.

Furthermore, two additional isolates were identified in the Yunnan province (China): W1V16, isolated from a single fecal sample of *Rhinolophus sinicus* (see Box 2), and RaTG13 from fecal samples of *Rhinolophus affinis*^25,26,32^. Further testing by infection of HeLa cells expressing humans, civet cats, and Chinese horseshoe bats ACE2 receptors, confirmed that these isolates could use them as cell entry mechanisms^27^. Cell susceptibility testing using different cell lines further indicated that WIV16 had the same host range as WIV1^25,27^. The ability of these strains to use ACE2 as entry receptors argued against the necessity for them of an intermediate host to reach humans^25,27^. This hypothesis was further supported by serological studies on human subjects who had previously been in contact with bats.

During a serological surveillance on 218 serum samples from residents in four villages in Jinning County (Yunnan province, China), Wang et al.^33^ found six individuals positive to CoV antibodies. A virus neutralization test targeting WIV1 and WIV16 was performed for the six positive samples^33^, and none were able to neutralize either virus, suggesting that the infection in the six subjects had been caused by a SARS-like-CoV virus variant less virulent than SARS-CoV^33^. The failure of the positive sera to either neutralize the virus, or to react with RBD proteins in Western blots could have been caused by the weakness of the response to the bat SARS-like-CoV S protein, or to the circulation of other bat SARS-like-CoV variants in these villages that have highly divergent S proteins^33^. The individuals found positive to the test lived between 1 and 6 km from two caves inhabited by bats of the genus *Rhinolophus spp*., a major reservoir of SARS-like-CoVs^33^. This region had not been involved in the 2002–2003 SARS outbreaks, and these individuals had not been previously in contact with areas, or patients, affected by SARS, suggesting that the positive serological tests were not due to prior infection with SARS-CoV^33^. The six seropositive subjects did not recall any clinical symptoms in the twelve months prior to the test, suggesting that the bat SARS-like-CoV infection had either occurred before the time of sampling, or that infections had been sub-clinical, and had caused only mild symptoms^33^. However, considering that these individuals had a high chance of direct exposure to bat secretion in their villages, it appears very likely that some bat SARS-like-CoVs had been able to directly infect humans without intermediate hosts, as suggested by the receptor entry and animal infection studies^26^.

An additional study, that directly supported the transmission of the coronaviruses to humans without the intervention of intermediate hosts, was performed by Wacharapluesadee et al.^34^. They identified HKU1 betacoronavirus in guano of bats in Thailand, and detected its presence in a non-hill individual with high level of occupational exposure to guano. The authors of the study reported that the contamination of the individual with the HKU1 virus was likely to have occurred through a human-to-human transmission rather than through exposure to bats, underlining the high risk of zoonotic spillover to people collecting guano^34^.

All considered, the possibility that the current epidemic of COVID-19 occurred in humans without the obligatory intervention of an intermediary animal species, appears highly plausible. Study on the genome sequence in COVID-19 found 96.2% identity with a previous SARS-like-CoV, the RaTG13^25,32^. Like the RaTg13, the novel coronavirus was able to use ACE2 receptors as cell entry mechanism, confirming that also the new coronavirus had a bat origin^32^. According to Andersen et al.^35^, a direct spillover of ancestors of the novel coronavirus might have been possible by acquiring the current genomic features through adaptations during undetected human-to-human transmissions. In fact, all the COVID19 genomes so far sequenced have the same features described above, indicating that they might have originated from a common ancestor^35^. If COVID-19 had pre-adapted in another animal species, then there would be a risk of future re-emergence events. In contrast, if the adaptive process had occurred in humans, the repeated zoonotic transfers would be unlikely to take off without the occurrence of the same series of mutations^35^: a time interval between different outbreaks would be expected, reflecting the time for the virus to adapt from the reservoir to the human host. It would seem an unlikely option, considering that COVID-19 had already well adapted to human host, with a very successful human-to-human transmission that allows the virus to be maintained within the population. Humans could thus be considered to have become the reservoir of COVID-19, eliminating the need of additional spillover from animal to human to keep the infection going.

Box 2 **SL-CoVs**

The SARS-like-CoVs has genome sequence identity of 88–90 % among themselves and 87–92 % to human or civet SARS-CoV isolates. The unique set of ORFs exclusively found in SARS-CoV was also present in bat SARS-like-CoVs, demonstrating the close phylogenetic relationship between SARS-CoV and SARS-like-CoV. SL-CoVs were also discovered in rhinolophids from Slovenia, Bulgaria and Italy in Europe but these European SARS-like-CoVs exhibited significant genetic variation from Chinese isolates^62,66^. In Africa, novel betacoronaviruses related to SARS-CoV have been detected in *Hipposideros* and *Chaerophon* bat species from Ghana, Kenya and Nigeria. However, compared with Asian and European SARS-like-CoVs, these viruses of non-rhinolophid origin were phylogenetically distant to SARS-CoV. The Western African isolates even formed a potential new lineage of Betacoronavirus in the phylogenetic tree^67,68^.

**WiV16**

The gene organization of WIV16 is identical to that of WIV1 strain, and slightly different from that of the civet SARS-CoV and other bat SARS-like-CoVs due to an additional ORF, ORFx detected between the ORF6 and ORF7 genes of the WIV1 and WIV16 genomes 27. The overall nucleotide sequence of WIV16 has 96% identity (higher than that of any previously reported bat SARS-like-CoVs WIV1) to human and civet SARS-CoVs^25,29,66,69^. The high sequence conversation of the WIV16 RBD with that of SARS-CoVs predicts that WIV16 is likely also to be able to use ACE2 as a cellular entry receptor. SL-WIV16-CoV was thus considered the closest ancestor to date of SARS-CoV and the study has provided further evidence that WIV16 is not the closest strain to the human SARSCoVs with regard to ORF8. Full-length ORF8 is present in several SARS-CoV genomes of early-phase patients. all civet SARS-CoVs, and bat SARS-like-CoVs. Furthermore, two studies have found a full-length ORF9 which has higher similarities to SARS-CoV GZ02 and civet SARS-CoV SZ3, suggesting that SARS-CoV derived from a complicated recombination and genetic evolution among different bat SARS-like-CoVs^70,71^.

## Civet cat and pangolin: the unlikely scapegoats of the two epidemics

During the SARS epidemic in 2002–2003, a new CoV was isolated from two clinically normal wild animal species from a wet-market in Shenzen (China): Himalayan palm civets (*Paguma larvata*), and a raccoon dog (*Neyctereutes procyonoides*). The virus was classified as member of the new SARS-CoV group^36^. The highest IgG antibody titers to SARS-CoV were observed in traders of masked palm civets (72.7%) compared to traders of all live animals (13%), and healthy controls (1.2%), concluding that the virus was transmitted by the animals from the wet-market^37^. Later, between December 2003 and January 2004, several new cases of SARS-CoV re-emerged in humans in the Guangdong Province (China), where most of them were not linked to civet cats^38^. Nonetheless, local government ordered the destruction of a large number of civet cats in the wet-markets, an action which was believed to have played a major role in the containment of re-emerged SARS-CoV, as no further cases were detected^38–40^.

However, subsequent extensive epidemiology studies failed to find SARS-CoV in farmed and wild-caught civet cats, indicating that other animal(s) might have been involved as the natural virus reservoir of SARS-CoV^41,42^. In addition, despite the high similarity between the receptor binding domains (RBD) in palm civet and human strains, which differ by only four residues, the affinity of the spike proteins for the human ACE2 receptor varies by more than 1000 fold^28^. In fact, strains of SARS-CoV isolated from palm civets in 2002–2003, and 2005 had low affinity for human ACE2 receptors and low infectivity in human cells, but had high affinity for civet cat ACE2 receptors and high infectivity in civet cat cells^36^. Therefore, these animals were evidently only incidental hosts, as there was no evidence for the circulation of SARS-like-CoV viruses in palm civets in the wild or in breeding facilities^43^. Currently, no intermediate host has been yet confirmed for SARS-CoV.

Something similar happened during the current COVID-19 epidemic. At the beginning of the epidemic a novel coronavirus which was considered highly related to COVID-19 was identified in Malayan pangolin (*Manis javanica*)^44^. It was proposed that the pangolins were potential natural intermediate hosts or even reservoirs of COVID-19,suggesting that these small mammals were a threat to public health^44^. Further studies, however, disproved this hypothesis. An analyzes of the genomes of the coronavirus identified in three sick pangolins found that the pangolin CoV is genetically related to COVID-19, but phylogenetic analysis and amino acid sequence on the S protein of COVID-19 did not support the hypothesis of the virus arising directly from the pangolin-CoV^45^. In addition, the recent work of Damas et al.^46^ examined ACE2 receptors from 410 vertebrates (fishes, amphibians, birds, reptiles, and mammals) to predict their ability to bind COVID-19 spike proteins. The vertebrates were classified as having *low*, *medium*, and *high* score depending on the ability of their receptors to bind COVID-19 Spike proteins^46^. The 18 species predicted as very *high* were all Old World primates and apes, which were identical to human in the 25 ACE2 receptor binding residues. Domestic cat scored *medium*, dog and pig scored *low*, while species scoring *very low* included all pangolin species such as Chinese pangolin (*Manis pentadactyla*), Sunda pangolin (*Manis javanica*), white-bellied pangolin (*Phataginus tricuspis*), and masked palm civet (*Paguma larvata*)^46^.

In addition, a recent study that investigated the possible role of pangolins as a source of potential zoonoses further exonerates the small mammal as potential intermediate host of COVID-19^47^. Throat and rectal swabs were collected from 334 Sunda pangolins (*Manis javanica*) confiscated and rescued from the wild in Peninsular Malaysia, and Sabah between August 2009 and March 2019^47^. No sample yielded a positive PCR result for any of the targeted viral families—Coronaviridae, Filoviridae, Flaviviridae, Orthomyxoviridae, and Paramyxoviridae^47^. The authors suggested that the lack of coronavirus detection in their samples might suggest that the previous detection of the virus in these animal species could reflect their exposure to infected humans, wildlife, or other animals within the wildlife trade network^47^. Thus, pangolins as civet cats could be considered only incidental hosts of coronaviruses.

Bats are common species in the wet-markets across China, and they are supplied by local poachers. For example, on February 28th 2020, 33 bats were confiscated from a poacher by the police in the Sichuan province^48^. The list of animals sold at Huanan market (the place considered the starting point of the epidemic in Wuhan) included different birds, reptiles, insects, and mammal species, among them bats, masked palm civet, badger, and foxes^49^. Bats are not a protected species in China. A study performed by Zhang et al.^50^ indicated a 60% decline in the bat population in the last 30 years in China, mainly caused to bat cave exploitation for tourism, and poaching by locals. The work of the poachers might have been important in the direct spillover of the pathogens from animals to humans, and in carrying the virus to different places. For example, a phylo-epidemiological study carried out by the Centre of Integrative Conservation (Xishuangbanna Tropical Botanical Garden) indicated that the COVID-19 found at Huanan market (Wuhan) might have not originated there, but it could have been imported from other places^51^.

Moreover, it must be kept in mind that wet-markets are also places where a large number of domestic animals are held such as pigs, cattle, dogs, cats, chickens, and ducks. Most of the time, the animals are slaughtered directly at the market. This could constitute a possible factor of contamination of the meat products with biological material coming from wild animals such as bats, or from infected humans. In fact, in a survey conducted on 242 subject form six different animals species in 2005, two pigs were found positive to SARS-CoV, while the owners of the animals were negative to the test^52^. The source of the SARS-CoV found in these pigs was most likely virus-contaminated animal food, since swineherds in rural areas often obtain leftovers from restaurants in the cities to be used for the pigs^52^. These findings might also explain the very recent COVID-19 outbreak in the biggest seafood market of Beijing, where over 100 people were found infected with the virus. All of them were workers of the Xinfadi market^53^. The animal products in that market were tested positive with COVID-19, including the wooden boards where the workers cut the meat^53^. The suggestion was made that the source of the infecting COVID-19 virus could have been imported salmon, that was keep frozen during the transport to Beijing, and could thus be contaminated with still alive virus. This new outbreak supports the hypothesis of contamination via animal products and not just via live animals. It is thus possible that crowded markets could function as boosting places for the pathogens. In fact, to go back to the *amplification effect*, people working at the wet-markets could be sensitive hosts in constant contact with the source of the virus. Moreover, the same workers of the market might slaughter the bats on the same area where they cut the meat contributing to further contamination. It is well known that wooden board can keep biological material even after been washed. In this environment, the virus would have all the time to adjust to the new host, maybe causing small undetected outbreaks until it reached the human-to-human form. In this situation, human beings would become the intermediate hosts of their own species.

## The pandemic stage: Wuhan and beyond

**Fig. 5:**
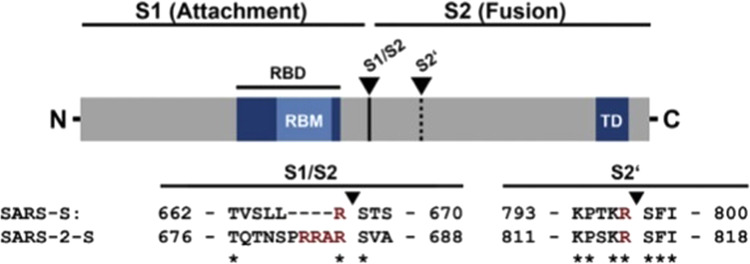
The proteolytic cleavage site in the S-protein of the SARS-CoV and SARS-CoV-2 viruses. The protease recognition site (the arginines, R) is different in the two viruses. The arrows indicate the cleavage sites (modified from Hoffmann et al.)^20^.

In the previous chapters, the discussion on the epidemics generated by SARS-CoV and COVID-19 has emphasized their important similarities. However, the two epidemics also have evident differences, the most obvious been the fact that SARS-CoV had somehow failed to reach the pandemic stage. It has now become clear that molecular aspects are likely to have had a role in it. The critical factor appears to be the difference in the S1–S2 cleavage in COVID-19 and SARS CoV, which could influence the efficacy of their interplay with the ACE2 receptor, and thus the infectivity of the two viruses^20^. The S1–S2 cleavage site of COVID-19 has several arginine residues, which indicate high cleavability, while that of SARS-CoV has only one. As a result, the S protein of COVID-19 is efficiently cleaved, but at this site that of SARS-CoV is not. However, the cleavage at the accessory S2’ site within the S2 subunit is instead the same in the two viruses^20^ (Fig. 5).

It should have become clear from the discussion above that the COVID-19 infection was present in China well in advance of January 2020, when it initiated its sudden path to the pandemic in Wuhan. The explosive growth after January of this year appears to have been made possible by the unfortunate combination of factors that have generated the *amplification effect* which is favored by the conditions prevailing and acting at their maximum in the wet markets of Wuhan. Very likely, they were potentiated by the fact that much larger viral loads could be exchanged in the man-to-man transmission in the wet market environment than in the pre-pandemic stage. However, even if the wet market in Wuhan has played a key role, cases of COVID-19 might have already occurred elsewhere prior to the explosion of the infection at the end of January, and they had apparently not been related to it: (1) on November 17th^,^ 2019, a 55 years old person from Hubei, who had not visited the Huanan market, was admitted to the hospital in Wuhan with serious respiratory symptoms^54^; (2) on December 1st 2019, another patient from Wuhan was diagnosed to have been infected by a new type of pneumonia virus^55,56^; (3) on December 10th 2019, 41 people in Wuhan were hospitalized with the coronavirus pneumonia, but 13 of them did not have link to the marketplace^55^.

One thing, which is still unknown, is whether the virus mutation (see below), that has later become dominant outside China, has originated in China from the Wuhan cluster or elsewhere, possibly even outside China. The possibility that the COVID-19 infection had already spread to Europe at the end of last year is now indicated by abundant, even if partially circumstantial, evidence. The occurrence of an unusually high number of interstitial pneumonias had indeed been noticed in Northern Italy and France in November and December of last year. However, no specific tests had been performed, thus the evidence that those cases were caused by COVID19 was only presumptive. The hypothesis of the presence of COVID19 was more likely for the patients ill with pneumonia, who had been admitted to two French hospitals in Colmar and Mulhouse, in November and December of last year. Retrospective analysis of the thoracic scans of some of the patients had revealed cases with typical radiological symptoms of COVID-19 pneumonia (see Box 3).

Eventually, the presence of COVID-19 in France before the end of last year was conclusively documented in a patient hospitalized in Paris on December 27 with severe pulmonary symptoms^57^. The causative role of COVID-19 in his pneumonia was retrospectively documented by Rt-PCR tests on a stored respiratory sample. The patient, an Algerian who had not been in touch with Chinese nationals, may have been infected by his wife, who worked in a supermarket close to the airport, where international passengers were shopping.

**Fig. 6:**
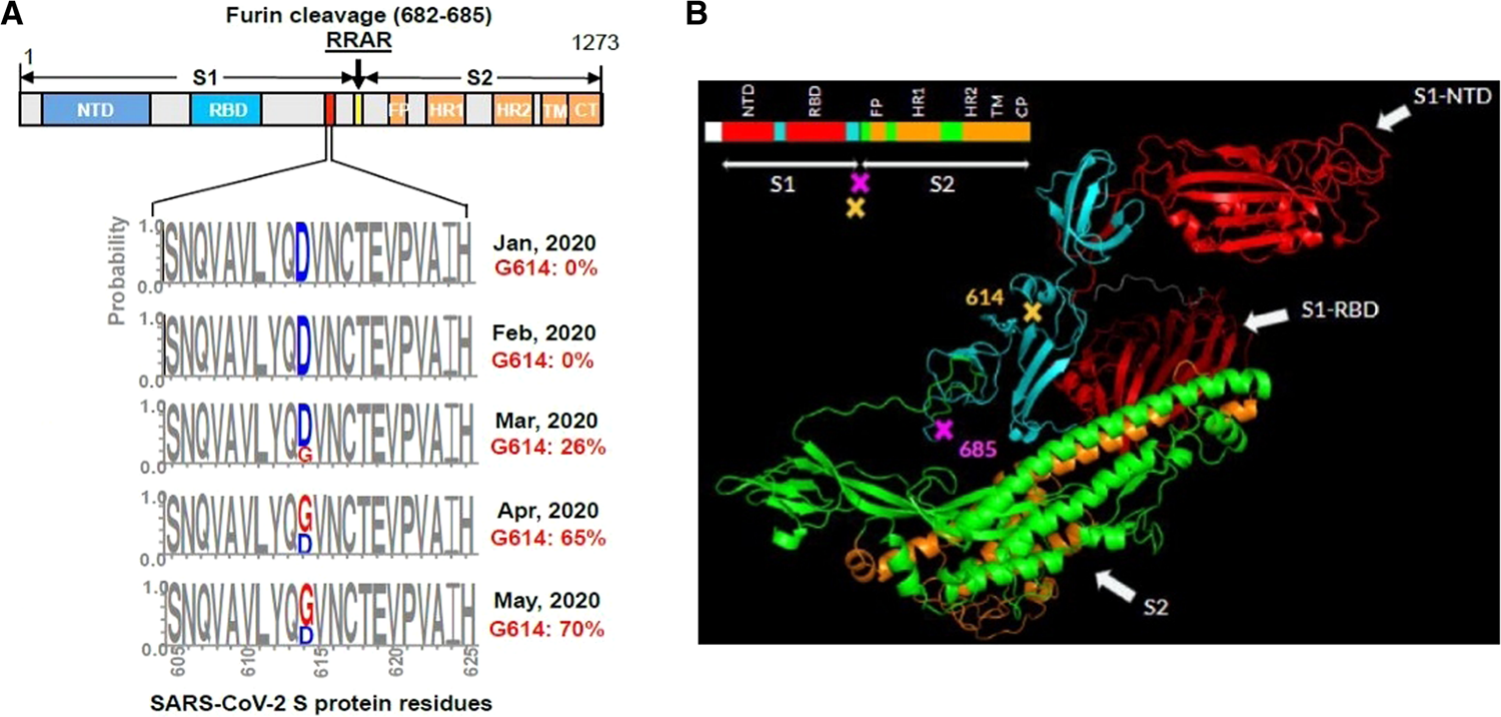
3-D structure protein S of the COVID-19. **a** The position of aspartate 614 is conserved among species, until the D-G mutation occurred in the virus variant now dominant in the European and American countries of the pandemic occurred. The D614G mutation generates an additional elastase proteolytic cleavage site that enhances viral membrane fusion next to the S1–S2 junction site (685) (modidfied from Bhattacharyya et al.)^60^. **b** Frequency of the D614/G mutation in the S-protein of COVID-19 in the S-protein sequences available in the GenBank from January to May, 2020. (modified from Zhang et al.)^61^.

To return to the present pandemic, one important fact that must be mentioned once more is that the early cases in Europe, and the localized outbreaks in China prior to the Wuhan outburst, had failed to spread epidemically. A number of reasons have been proposed to explain the finding. For example, in those initial episodes, the virus had not been permitted to spread to an adequately large number of individuals such as the infected people in remote villages close to the bat caves in China, and the patient who was immediately hospitalized in France. It has now become clear that the most important reason is the virus itself, which at that initial stage still had a relatively low transmission ability. As of today, about 160 complete genomes of COVID-19 have been sequenced from human patients: phylogenetic work on them has identified three central variants distinguished by aminoacid changes: A, B, and C^58^. A is the ancestral type, as it is the closest to that discovered in bats. The three variants have different geographical distribution, B being the most common in East Asia, and A and C outside East Asia, i.e., Europe and the Americas. More than 80,000 sequences of the virus are now available (the majority of them from COVID-19), and well over 100 mutations have been described. Among them, the most interesting is a D614G mutation in the C terminal region of subunit S1 of the Spike protein, which is the region in which subunit S1 associates with subunit S2 (Fig. 6a). It is still not clear whether this mutation has originated in East Asia or in Europe, but what is known is that the mutated virus has been found to spread with alarming speed outside East Asia over a period of a few months becoming the dominant form of COVID-19 in Europe and the Americas^59^. The form of the disease caused by the virus with the D614G mutation was first documented in a case in Germany in February 2019 (sequence deposited on January 28), and then the mutated virus spread across Europe to rapidly represent the dominant form of the COVID-19 pandemic. By the end of January, 98.3 % of the sequences submitted from East Asia and 90-to 100% of those submitted from the remainder of the world were of the “correct” subtype with an aspartate in position 614. By the end of February the frequency of the mutated subtype had already risen to over 50% in Europe, and about 10% in North America (Fig. 6b). By contrast, the non-mutated subtype has remained low in China and other East Asia countries^60^. The spread of the mutated sub-type in Europe and the American continent has then continued to grow explosively to more than 200 countries in different continents.

The mutation confers to the virus a decisive transmission advantage over the non-mutated variant: it was found that ACE2 receptor-expressing cells become infected much more efficiently by the mutated virus than by the wild type variant^61^. The D614/G mutation could promote an open configuration of the Spike protein that would favor the association with the ACE2 receptor. Therefore, an increased infection would be achieved by reducing S1 shedding and by increasing the total amount of the Spike protein incorporated into the virion^61^. The D614G mutation near the S1–S2 junction of the spike protein creates an additional vicinal proteolytic site for the serine protease elastase produced by the neutrophils. Sequential cleavages at the S1–S2 junction would give the virion additional advantage in the penetration into the host cell^62^.

At this point, a small digression affords a comment of general nature on the matter of the spreading of coronavirus infections. It appears that the D614G mutation of the Spike protein (personal communication of Enrico Bucci: bucci@shro.org) is also present in SARS-CoV. In principle, based on what has happened with COVID-19, the mutation could have made the SARS-CoV infection capable of proceeding to a pandemic. But fortunately, in SARS-CoV the mutation was accompanied by the mutations in the S1-S2 cleavage site described above that limited its infectivity. One could consider it an abortive attempt of evolution to generate a coronavirus variant far deadlier than COVID-19.

Other factors not directly related to the properties of the virus could still have had a role in the generation of the clusters of high infection density that have been instrumental in the rapid globalization of the pandemic. One important factor has certainly been the magnitude of the viral load that had assailed the various “*patients zero*” of each cluster. Another factor that could have played a role might be in all likelihood the patient himself, i.e., its “physiology”: For instance, just to name one possibility, the amount of ACE2 receptors he expresses. Last but not least, another factor to be considered is the social attitude of the infected person that determines whether he interacts with an adequately large number of subjects.

Box 3 Of special interest for the present discussion on the pre-pandemic phase are the case of athletes that had participated in the World Military Games that took place in Wuhan from October 18 through October 27, 2019. The event had brought to Wuhan more than 10,000 athletes, plus a very large number of staff personnel: they had come from 110 Countries and of course from all of China as well. A number of them had developed symptoms typical of COVID-19 infection after they had returned home to several countries, e.g., Sweden, France, Italy, and the USA. Actually, five USA athletes had to be repatriated ahead of the end of the games with the diagnosis, but doubts are legitimate, since malaria is not a contagious disease.

## Conclusions

**Fig. 7:**
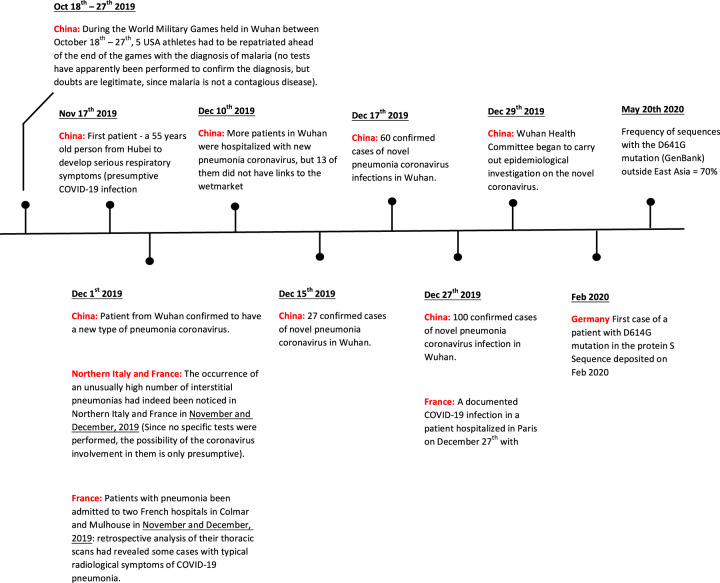
Timeline of the events in the 2020 COVID-19 pandemic.

The timeline of the origin and development of the present pandemic that emerges from the data and the discussions above is a suitable way to conclude the contribution (Fig. 7). It shows that the COVID-19 disease was present in China—and also in Europe—well in advance of its sudden explosion in Wuhan at the beginning of this year. Nevertheless, the disease had remained in a dormant state because the other factor which was essential for its vigorous awakening, the local *amplification effect*, was absent. In hindsight, it could be speculated that it was a matter of time: sooner or later, the explosion was bound to happen.

The contribution has discussed, and clarified when necessary, some other points which were still obscure in the general problem of COVID-19. One is the conclusion that in the travel from bat to human the virus did not necessarily need an intermediate host. Another is the reason why the epidemic of SARS-CoV had somehow failed to set off a pandemic of global dimension, and the reason why, by contrast, COVID-19 has instead been able to trigger the enormous increase of the transmissibility once it left its initial Chinese birthplace.

In closing, it seems appropriate to mention one last study, interesting but intriguing at the same time, that has appeared very recently^63^. Two genomic regions have been identified in humans which are associated with COVID-19 pathology: one on chromosome 3 that contains six genes, and one on chromosome 9 that determines the ABO blood group^64,65^. Only the first has been significantly associated with severe COVID-19 at the genome-wide level. The genetic variants associated with severe COVID-19 pathology on chromosome 3 span 49,000 bases. The study has analyzed various possibilities, and has concluded that the DNA haplotype could have entered the human population by hybridization- linked gene flow from Neanderthals. It occurs at a frequency of 30% in South Asia, of 8% in Europe, at 4% in Americans, and at lower frequencies in East Asia. It is not known how the Neanderthal-derived haplotype would confer COVID-19 risk and/or, for that matter, risk to other pathogens. Nevertheless, the study concludes that, in the present pandemic the “*gene flow from Neanderthals has tragic consequences*”.
